# Vascular Endothelial Growth Factor B and Its Signaling

**DOI:** 10.3389/fcvm.2018.00039

**Published:** 2018-04-20

**Authors:** Nathaniel Lal, Karanjit Puri, Brian Rodrigues

**Affiliations:** Faculty of Pharmaceutical Sciences, The University of British Columbia, Vancouver, BC, Canada

**Keywords:** STZ diabetes, VEGFB, angiogenesis, cell death, cardiomyopathy

## Abstract

In diabetes, compromised glucose utilization leads the heart to use FA almost exclusively for ATP generation. Chronically, this adaptation unfortunately leads to the conversion of FA to potentially toxic FA metabolites. Paired with increased formation of reactive oxygen species related to excessive mitochondrial oxidation of FA, can provoke cardiac cell death. To protect against this cell demise, intrinsic mechanisms must be available to the heart. Vascular endothelial growth factor B (VEGFB) may be one growth factor that plays an important role in protecting against heart failure. As a member of the VEGF family, initial studies with VEGFB focused on its role in angiogenesis. Surprisingly, VEGFB does not appear to play a direct role in angiogenesis under normal conditions or even when overexpressed, but has been implicated in influencing vascular growth indirectly by affecting VEGFA action. Intriguingly, VEGFB has also been shown to alter gene expression of proteins involved in cardiac metabolism and promote cell survival. Conversely, multiple models of heart failure, including diabetic cardiomyopathy, have indicated a significant drop in VEGFB. In this review, we will discuss the biology of VEGFB, and its relationship to diabetic cardiomyopathy.

## Introduction

The incidence of diabetes has reached epidemic proportions, with approximately 366 million people affected globally. Cardiovascular disease is the leading cause of diabetes-related death and this heart failure could be an outcome of atherosclerotic coronary artery disease or a consequence of an intrinsic malfunction of the heart muscle (labeled diabetic cardiomyopathy) (1–3). Diabetic cardiomyopathy is a complicated disorder and several factors have been associated with its development. These include an accumulation of connective tissue and insoluble collagen, impaired sensitivity to various ligands (e.g., β-agonists), mitochondrial dysfunction, ER stress, RAAS activation and abnormalities in proteins that regulate intracellular calcium (4). Additionally, changes in cardiac metabolism have also been reported in diabetic cardiomyopathy and are considered a principal culprit in its initiation. Metabolic changes embrace reduced glucose consumption, with a switch to predominant fatty acid (FA) utilization (5). Unlike glucose, the oxidation of FAs requires proportionally greater oxygen to produce a similar amount of ATP (6). Regrettably, augmented FA oxidation increases the generation of reactive oxygen species (ROS) which have been implicated in apoptotic cell death. Therefore, in the diabetic heart it would be useful to have arrangements to (a) promote angiogenesis (to ensure a steady supply of oxygen to metabolize this excess of FA), and (b) prevent cell demise (associated with increased FA oxidation). Members belonging to the vascular endothelial growth factor (VEGF) family of proteins are unique in their ability to modulate both oxygen delivery and inhibit programmed cell death (7).

### VEGF Family of Proteins

**FIGURE 1:**
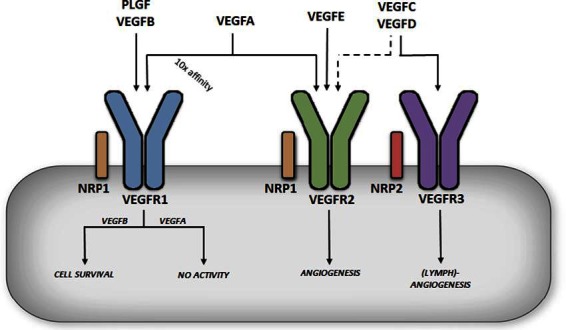
Differential functions of vascular endothelial growth factor receptors. VEGFA binding to VEGFR1 does not produce significant receptor activation (in this case the receptor acts a decoy), whereas VEGFB binding to VEGFR1 has been described to promote cell survival. VEGFA can also bind to VEGFR2, albeit with a lower affinity, but this is considered a key regulator of angiogenesis, promoting endothelial cell migration and proliferation.

The VEGF family consists of 6 growth factors; VEGFA, VEGFB, VEGFC, VEGFD, VEGFE and placental growth factor (PLGF) (8). These growth factors are able to bind and activate tyrosine kinase receptors called vascular endothelial growth factor receptors, of which there are three major types (VEGFR1-3) (Figure 1). VEGFA is able to bind VEGFR1 and VEGFR2, VEGFB and PLGF can only bind VEGFR1, VEGFC and VEGFD bind to both VEGFR2 and VEGFR3 while VEGFE only binds to VEGFR2. In addition to VEGFRs, there are two co-receptors, Neuropilin-1 (NRP1) and Neuropilin-2 (NRP2) (Figure 1). These co-receptors can bind to VEGFRs to potentiate the latter’s action; some VEGFs can also bind independently to NRPs (9).

### VEGFA

The most extensively studied member of the VEGF family, VEGFA, was first isolated and cloned in 1989 (10). The VEGFA gene is located on chromosome 6p21.1 in humans, contains 8 exons, and alternative splicing through exons 6 and 7 leads to a number of isoforms named after the number of amino acid left after cleavage of the signal peptide (11). VEGFA_121_ lacks both exons 6 and 7, which encode highly basic heparin binding domains, making this isoform acidic and freely soluble once secreted. VEGFA_186_, which lacks exon 6B, and the full length VEGFA_206_ contain multiple heparin binding domains allowing for almost complete sequestration once secreted, due to their high heparin affinity. The most abundant and biologically relevant isoform, VEGFA_165_ (henceforth referred to as VEGFA), lacks exon 6 but contains the heparin binding domain encoded in exon 7, allowing it to be sequestered onto the cell surface or extra cellular matrix once secreted. VEGFA is considered a key regulator of angiogenesis, promoting endothelial cell (EC) migration and proliferation (12) (Figure 1). This important role was illustrated in genetic studies where manipulation of this growth factor revealed severe defects in angiogenesis, with *VEGFA^ +/−^* mice dying *in utero* (13). VEGFA also exhibits a number of additional functions; through the regulation of endothelial nitric oxide synthase (eNOS), it can increase vascular permeability and vasodilation (14), whereas increased Akt signaling gives VEGFA a role in cell survival (15). It has also been implicated in monocyte chemotaxis (16) and colony formation (17). The functions of VEGFA are mediated primarily through binding and activation of VEGFR2. Interestingly, VEFGA also binds to VEGFR1 and that too, with a 10-fold higher affinity (18). Paradoxically, binding of VEGFA to VEGFR1 has limited receptor activation, suggestive of VEGFR1 being a negative regulator of VEGFA action (19) (Figure 1). In this regard, VEGFR1 knockout is embryonically lethal as in the absence of this receptor, VEGFA only binds to VEGFR2 leading to hyper vascularization (20).

### Other VEGFS

VEGFC and VEGFD play a prominent role in lymphoangiogenesis (21, 22). Both of these growth factors are secreted as pro-proteins with long C and N terminals that require cleaving for them to become fully active and bind to VEGFR2 and VEGFR3 (Figure 1). Deletion of VEGFD does not produce any obvious phenotype (23), while experiments with recombinant VEGFD promote lymphatic EC angiogenesis. Deletion of VEGFC is embryonically lethal due to lack of lymphatic vessel development (24) while overexpression of VEGFC results in selective induction of lymphatic EC proliferation (25). VEGFE was discovered in the genome of the Orf virus which can occasionally infect humans through contact with goats and sheep, and leads to highly vascularized skin lesions (26). VEGFE acts in a similar fashion as VEGFA_165_ but only binds VEGFR2 (Figure 1) and is structurally similar to VEGFA_121_ with no heparin binding capabilities. PLGF is structurally similar to VEGFA, containing four isoforms (PLGF1-4) (27). PLGF1 and PLGF3 are freely diffusible lacking a heparin binding domain, while PLGF2 and PLGF4 contain an additional 21 basic amino acids enabling these isoforms to be sequestered. PLGF can only bind VEGFR1 but unlike VEGFA, PLGF is pro-angiogenic on binding to this receptor (Figure 1). PLGF null mice are healthy indicating PLGF as being redundant for vascular development. However, knockout of PLGF shows impaired angiogenesis in pathological conditions such as ischemia (28). For additional information regarding the above VEGF family members, the reader is directed towards these excellent reviews (8, 12, 29, 30). The current review will focus on VEGFB.

## VEGFB

### Overview

**FIGURE 2:**
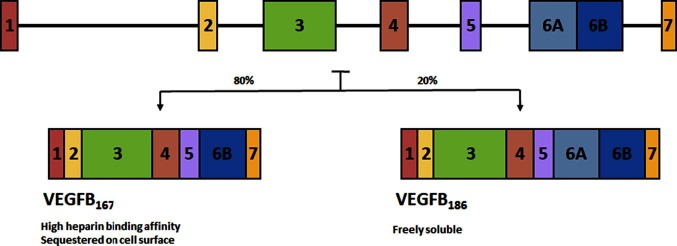
Alternative splicing of VEGFB. Alternative splicing leads to two isoforms. VEGFB_167_ encompasses over 80% of transcripts and contains a highly basic C-terminal heparin binding domain allowing it to be sequestered onto the cell surface. The other isoform, VEGFB_186_ has a hydrophobic C-terminal making it freely soluble.

The VEGFB gene is located on chromosome 11q13.1 and alternative splicing leads to two isoforms. VEGFB_167_ encompasses over 80% of transcripts (31) and contains a highly basic C-terminal heparin binding domain allowing it to be sequestered onto the cell surface, much like VEGFA_165_. The other isoform, VEGFB_186_ has a hydrophobic C-terminal making it freely soluble (32) (Figure 2). Tissue expression analysis of VEGFB observed this growth factor to be highly expressed in the heart and skeletal muscle, with limited expression in most other tissues (33). The genetic expression of VEGFB is fairly stable and is not regulated by growth factors, hypoxia or hormones (34). Genetic knockout of VEGFB demonstrated that this growth factor is not relevant for normal health (35), as VEGFB null mice developed only a mild phenotype with no effect on mortality.

### Function

**FIGURE 3:**
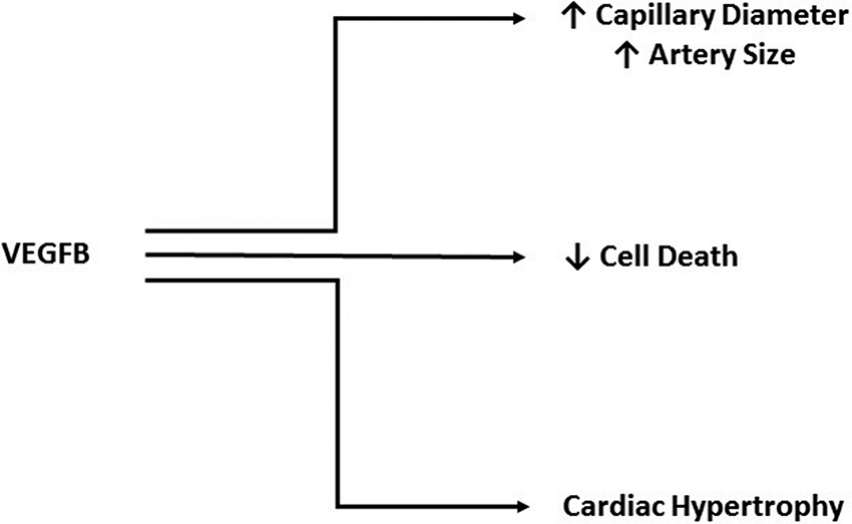
Cardio protective actions of VEGFB. Although it does not play a direct role in angiogenesis, VEGFB has been implicated in increasing capillary diameter and artery size. Additionally, VEGFB has a significant role in protecting against cell death and promoting adaptive hypertrophy.

VEGFB shares 47% of its amino acid sequence with VEGFA and contains the hallmark PXCVXXX-RCXGCC VEGF family motif (33), which lead to the initial studies focused on a role for VEGFB in angiogenesis. Unlike the other members of the VEGF family, VEGFB uncharacteristically does not promote angiogenesis (36–38). For example, although it was suggested that VEGFB is able to induce EC growth, this was ultimately attributed to VEGFA/VEGFB heterodimer formation, and not a direct function of VEGFB (39). Through the use of adenoviral vectors to promote overexpression in muscle or peri-adventital tissue, VEGFB was unable to stimulate vessel growth (40). In mice transgenically overexpressing VEGFB in the skin, there was limited effect on blood vessel density although an increase in capillary diameter was observed (41). Finally, ischemic limb studies provided additional evidence against VEGFB being an angiogenic growth factor as it was ineffective in aiding vascular growth (42). Due to an absence of a role in angiogenesis, the novelty of this growth factor diminished after its discovery. More recently, the importance of VEGFB has been described in cell survival (38) (Figure 3), a function that is especially relevant under pathological conditions (43), and will be discussed later.

### Receptor Binding

The action of VEGFB is coordinated by its binding to VEGFR1. The VEGFR1 gene is located on chromosome 13q12.3 and encodes a tyrosine kinase receptor comprised of an extracellular ligand binding domain, a transmembrane domain, intracellular tyrosine kinase domain and a carboxy-terminal region (18). Alternative splicing also generates a soluble form of VEGFR1 (sVEGFR1) that only contains the extra cellular ligand binding domain. Three members of the VEGF family, VEGFA, VEGFB and PLGF can bind VEGFR1, each with distinct functions. VEGFB binding leads to activation of a number of downstream activators similar to most tyrosine kinase receptors, including p38 MAPK, ERK/MAPK, PKB/AKT and PI3K (44). VEGFA binding to VEGFR1 does not produce significant receptor activation (44) (Figure 1), indicating that VEGFR1 could act as a negative regulator of VEGFA action. In this regard, deletion of the VEGFR1 gene is embryonically lethal (45), likely due to enhanced VEGFA action on VEGFR2, promoting uncontrolled angiogenesis. Interestingly, deletion of only the tyrosine kinase domain results in normal healthy mice (46), suggesting that the ligand binding domain is necessary for VEGFR1 regulation of VEGFA. Further evidence for a role of this receptor in regulating proper angiogenesis is the expression of this receptor primarily in the blood vessels of numerous organs such as the heart, kidney, liver and brain (47). Soluble VEGFR1 is also a potent anti-angiogenic factor that can bind VEGFA in the plasma. PLGF will bind VEGFR1, but unlike VEGFB, can induce angiogenesis (48) (Figure 1). The effect of binding of PLGF to VEGFR1 to induce angiogenesis has been suggested to be a consequence of its interaction with the immunoglobulin domains two and three of the receptor, an action that is not seen with VEGFB that only binds to Ig domain two (49).

### The Action of VEGFB to Influence Angiogenesis

**FIGURE 4:**
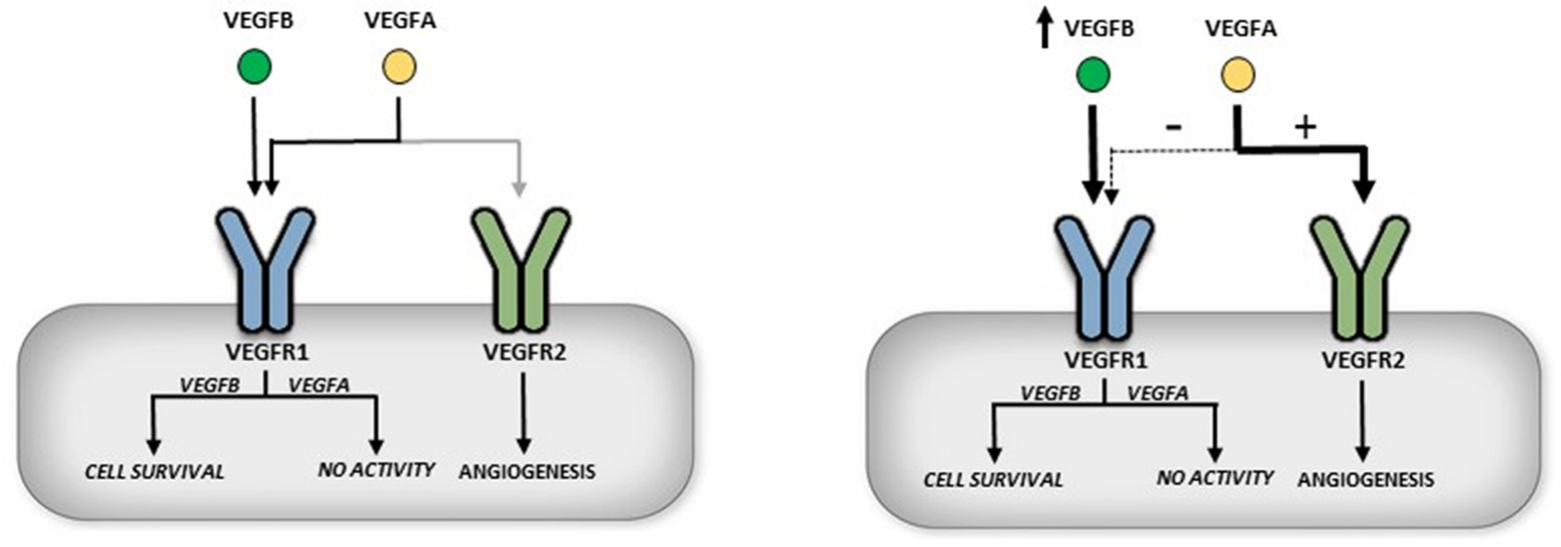
Indirect role of VEGFB in angiogenesis. VEGFR1 has a 10× fold greater affinity for binding VEGFA than VEGFR2 (left panel). Overexpression of VEGFB is suggested to occupy and thus displace VEGFA from VEGFR1. This allows more VEGFA to bind to VEGFR2 and can initiate angiogenesis (right panel).

Unlike the futile studies related to examining the function of VEGFB in angiogenesis, recent studies have recognized a prominent role of this growth factor in sensitizing cells to VEGFA induced angiogenesis (43, 50). This phenomenon can be explained through the specificity of VEGFA and VEGFB binding to VEGFR1 and VEGFR2. VEGFA can bind to both VEGFR1 and VEGFR2, but only by binding VEGFR2 does VEGFA activate downstream signaling. It should be noted that VEGFR1 has an order of magnitude greater affinity for VEGFA (18). Conversely, binding of VEGFB to VEGFR1 initiates downstream signals (Figure 1). Transgenic (TG) overexpression of VEGFB revealed an interesting mechanism in which excess VEGFB can occupy VEGFR1, allowing more VEGFA activation of VEGFR2 (Figure 4). In a cardiomyocyte specific overexpression of VEGFB, TG rat hearts displayed enhanced activation of multiple downstream signaling VEGFA targets (43). Moreover, VEGFA administration to VEGFB TG animals presented greater downstream signaling after 10 min compared to wildtype. Accordingly, VEGFB knockout animals demonstrated blunted VEGFA action compared to wildtype, likely a result of increased available VEGFR1 negatively regulating this growth factor. In a more recent study, adeno-associated virus (AAV) VEGFB transduction in mice showed unanticipated vascular effects in adipose tissue (50). As quickly as two weeks post AAV administration, increased capillary density and vessel size was observed. This enhanced capillary network displayed a normal pattern compared to AAV administration of VEGFA which revealed abnormal vasculature and increased infiltration of inflammatory cells. This study reasoned that the increased vasculature seen in VEGFB AAV animals was not due to a direct effect of VEGFB but a result of increased VEGFA action of VEGFR2 due to less available VEGFR1 (Figure 4).

### Genetic Manipulation

In addition to these indirect effects of VEGFB in enhancing VEGFA action, recent studies have recognized a prominent role of this growth factor in cell survival, and a number of exciting areas of research have emerged. While knockout of the VEGFB gene has limited consequences, employing this mouse model in various disease conditions has uncovered a protective role for VEGFB. In a cerebral ischemic injury model, VEGFB^−/−^ mice displayed a 40% greater increase in infarct size as well as severity of brain dysfunction compared to wildtype animals (51). This study also examined cultured neurons exposed to hypoxia to induce cell death and determined that cells cultured with 100 ng/mL VEGFB had less demise, further reinforcing a neuroprotective role for VEGFB. Through the use of a mouse cornea pocket assay which causes degradation of corneal blood vessels, VEGFB null mice displayed accelerated degeneration and after three weeks had fewer blood vessels (38). Furthermore, in an oxygen induced blood vessel regression model, VEGFB deficient mice had increased regression and treatment with a VEGFB neutralizing antibody further intensified this observation. Conversely, intravitreal VEGFB treatment inhibited blood vessel regression. This survival effect of VEGFB was also observed in cells other than vascular EC. Hence, when primary chordial EC, retinal EC, CD133^+^CD34^+^ stem cells and aortic smooth muscle cells (SMC) isolated from VEGFB null mice were cultured in serum free medium or under H_2_O_2_ induced stress, they exhibited increased apoptosis and VEGFB treatment of these cells reduced this effect. Culture of retinal EC, chordial EC, pericytes, and SMC immortalized cell lines also show decrease serum starved cell death when treated with VEGFB (Figure 3). Finally, in a model of acute myocardial infarction, VEGFB^−/−^ mice demonstrated reduced revascularization of the ischemic border 7 days post MI as a consequence of fewer thrombomodulin positive capillaries and smooth muscle α-actin positive covered vessels in the infarct area compared to wildtype animals (42). As was seen in other studies and models, administration of VEGFB to these VEGFB^−/−^ mice induced revascularization.

Unlike VEGFB knockout animals, TG overexpression of VEGFB (particularly in the heart) produced significant alterations that were cardio protective. Rats with the VEGFB gene attached to the αMHC promoter generates a cardiomyocyte specific overexpression of VEGFB. These animals displayed a robust increase in arteries of all sizes, especially vessels >150 µm, in which there was a five-fold increase. Additionally, these hearts had capillaries with larger diameters (43) (Figure 3). A unique feature of these TG hearts was that they exhibited cardiac hypertrophy, but this was not pathological as even at 22 months, there was no difference in ejection fraction, fractional shortening or maximal exercise capability. Moreover, gene expression analysis identified no change in pathological hypertrophy genes. Exposing these animals to experimental MI revealed marked differences between the groups. Echocardiography showed a less severe decrease in ejection fraction, fractional shortening and an increase in left ventricular systolic and diastolic diameters in TG rats, when measured at both 1 and 4 weeks post MI. The TG hearts also demonstrated better perfusion in both the non-infarcted area as well as the infarcted and border areas. Analysis of the infarct size post-mortem confirmed a substantial decrease in infarct size in TG hearts from both male and female rats. Additionally, this study utilized a 2 week angiotensin II treatment to model pathological hypertrophy and found decreased VEGFB. Along similar lines, in human heart samples from patients that underwent heart transplant, there was decreased VEGFB mRNA in hearts from patients with ischemic heart disease or dilated cardiomyopathy compared to donor hearts that were not used for transplant (43). In another human study, patients that had suffered an acute myocardial infarction (AMI) displayed increased plasma VEGFB compared to healthy volunteers (52). However, within the AMI group, those patients that were on the lower scale of the plasma VEGFB spectrum prior to discharge, exhibited increased left ventricular remodeling six months post MI, a marker for potential left ventricular dysfunction and heart failure. These studies have led to investigation of VEGFB gene therapy in a number of heart disease models. In one study, mice that underwent transversal aortic constriction displayed decreased ejection fraction and fractional shortening, along with left ventricular hypertrophy and decreased VEGFB mRNA four weeks after surgery compared to sham treated animals (53). Treatment via VEGFB viral vectors 2 weeks post-surgery abolished the decreases in ejection fraction and fractional shortening; animals also displayed less severe hypertrophy. These effects were suggested to be due to an increase in cardiomyocyte proliferation (detected by Ki-67 immunostaining) and decreased apoptosis (seen via cleaved caspase 3 immunostaining). In another study which used VEGFB_186_, dogs were exposed to 28 days of left ventricular pacing via an external pacemaker to induce a model of dilated cardiomyopathy (54). Intracoronary VEGFB_186_ was delivered either 2 days prior to pacing or 2 weeks after initiation of the pacing protocol (these animals were labeled delayed AAV-CMV-VEGFB). Paced animals that did not receive VEGFB_186_ transgene displayed typical signs of decompensated heart failure with increased left ventricular end-diastolic pressure, decreased left ventricular systolic pressure and decreased mean arterial pressure. Animals given VEGFB_186_ viral vectors prior to pacing showed no significant changes while the delayed VEGFB_186_ treated animals had significant changes after 2 weeks of pacing but no further changes once VEGFB treatment was initiated. Finally, the cardio-protective ability of VEGFB was examined with respect to mitigating the cardio toxic effects of drugs like doxorubicin (55). Doxorubicin is a commonly used anti-cancer drug that is effective against a variety of cancers by inhibiting cell cycle progression and stopping proliferation of malignant cells (56). However, multiple doses of doxorubicin have been found to be cardio toxic, leading to left ventricular dysfunction and heart failure. Mice injected with AAV9-VEGFB seven days before initiation of a multiple doxorubicin dose protocol, saw no decrease in heart weight and cardiomyocyte size as seen in control mice (55). Furthermore, VEGFB pretreatment prevented cardiac microvasculature damage. Additionally, a single high dose injection of doxorubicin induced DNA double-strand breaks which was reduced in VEGFB pretreated animals.

In contrast to the genetic studies with VEGFB suggesting a role for this growth factor in cell survival, genetic manipulation of VEGFR1, particularly sVEGFR1, is seen as an anti-angiogenic cancer therapy (57). Anti-VEGF treatments continue to be heavily investigated in cancer therapy due to the hypoxic conditions found in most tumors leading to enhanced VEGF action. As outlined previously, VEGFR1 has an interesting interaction with VEGFA; this receptor has a 10 times greater affinity for VEGFA compared to VEGFR2 but produces minimal downstream signals (18). Soluble VEGFR1 also has this unique property for VEGFA binding, as well as being freely diffusible in the circulation to take up any excess VEGFA, limiting tumor angiogenesis. When tumor cells were transfected with sVEGFR1 cDNA and injected into nude mice to observe tumor growth, cells that had higher sVEGFR1 expression grew much slower than unaffected tumor cells (58). In a different experiment, animals injected with tumor cells expressing more sVEGFR1 lived nearly twice as long as control.

### Protective Cell Survival Role of Recombinant VEGFB

In addition to the genetic studies examining the effects of VEGFB on cell survival, the use of recombinant VEGFB protein has also been employed to investigate the mechanisms of this growth factor in preventing cell death (Figure 3). Treatment of primary aortic SMC with human recombinant VEGFB downregulated many genes involved in apoptosis such as *Bmf, TrP53inp1*, and *DCN (59)*. In cell lines treated with VEGFB, there was a substantial downregulation of many BH3-only protein genes such as *Bad* and* Bid,* and other genes related to cell death like *Casp9, Bax* and *TNF-α*.

A rat ganglion cell line treated with VEGFB and exposed to hydrogen peroxide (H_2_O_2_) or serum starvation to induce cell death revealed that VEGFB was protective in both instances and promoted cell survival (59). This serum starved cell survival effect was not seen with other VEGFR1 ligands like PLGF, whereas VEGFA had a weaker effect. Additionally, in an optic nerve crush injury model, injection of VEGFB into the eye following injury resulted in an increase in the number of viable ganglion cells when measured 2 weeks post procedure. Similarly, inhibition of VEGFB with a neutralizing antibody decreased the number of viable cells in this model. VEGFB treatment was also able to reduce neuronal apoptosis following NMDA or ischemia induced apoptosis. In all of these models, real-time PCR revealed that VEGFB treatment reduced the expression of apoptotic genes and VEGFR1 blockade using a neutralizing antibody eliminated the protective effects of VEGFB, implying that the benefits of this growth factor are found by binding VEGFR1 (59). In another study, cardiomyocytes were exposed to 48 h of hypoxia and then 24 h of reoxygenation with or without VEGFB. The presence of this growth factor decreased the percentage of apoptotic cells and a similar finding was seen with cells treated with the cardiotoxic drug epirubicin (60). VEGFB treatment of cardiomyocytes for 24 h increased the expression of many genes involved in contractility (*αMHC*), calcium handling (*SERCA2a*) and mitochondrial function (PGC1α) in a manner similar to that seen with compensatory hypertrophy induced by the thyroid hormone T3 (Figure 3).

### VEGFB in Cancer

Many pro survival factors that have beneficial effects in numerous disease and injury conditions have unfortunately also been implicated in other pathologies, and VEGFB is no exception. VEGFB expression can be found in many different tumor types and its expression has been found to be increased in multiple cancers such as renal carcinomas and hepatocellular carcinomas (61). In examining patients with hepatocellular carcinomas, researchers found that higher VEGFB expression correlated with advanced stage, multiple tumors, positive vascular invasion and lack of capsule formation (62). Interestingly, a study found that gain of VEGFB function in a cancer cell line with low VEGFB resulted in an increase in circulating tumor cells and metastases (63). Enhanced VEGFB function led to reduced perivascular cells and increased vascular leakiness, inflammation, hypoxia and M2-like macrophages. These effects of VEGFB were independent of VEGFA as a cancer cell line that doesn’t express VEGFA as well as cells treated with a VEGFA neutralizing antibody still displayed increased circulating tumor cells with increased VEGFB expression. Implanting high VEGFB expressing tumors into mice lacking the tyrosine kinase domain of VEGFR1 did not entirely stop VEGFB induced metastasis, suggesting a possible VEGFR1 independent mechanism. VEGFR1 also has a prominent role in cancer and interestingly, like VEGFB, VEGFA can also signal through this receptor (64). Hence, treatment of the colorectal carcinoma cell line HT-29, (which does not express VEGFR2), with either VEGFA or VEGFB produced signal activation with increased phosphorylation of extracellular signal-regulated kinases 1/2 (ERK 1/2) and c-Jun N-terminal kinases (JNK). These treatments also lead to increased cell migration and invasion which was inhibited with the VEGFR1 neutralizing antibody 18F1. A similar study was done using a number of pancreatic carcinoma cell lines that expressed VEGFR1 but no VEGFR2 (65). VEGFA or VEGFB treatment lead to phosphorylation of ERK 1/2 but not JNK. Increased cell proliferation and invasion was seen with VEGFA or VEGFB treatment and inhibited by 18F1.

### VEGFB in Diabetes

As outlined in this review, VEGFB has been indicated to play a role in cell survival and indirectly promote VEGFA induced angiogenesis (11, 38, 43) (Figure 4). Both of these functions are highly desirable, particularly during diabetes, with increased cardiomyocyte demise and poor angiogenesis in the heart being hallmark conditions associated with this disease (66, 67). Currently it is unclear whether this increased cell death or decreased angiogenesis in the heart is an outcome of changes in VEGFB. We injected rats with streptozotocin (STZ), a β-cell-specific toxin to induce diabetes. A single dose of 55 mg/kg (D55) STZ was used to induce moderate diabetes. Our contention is that this D55 model of diabetes imitates the clinical phenomenon of insufficient glycemic management in T1D where multiple finger pricks and daily insulin injections (3–4/day) mean poor patient compliance and repeated exposure to bouts of acute hyperglycemia. These animals were kept for 6 weeks, a well-established model of diabetic cardiomyopathy. Analysis of cardiomyocyte VEGFB protein and mRNA expression revealed a significant decrease in the production of this growth factor (68). Furthermore, there was reduced cell survival signaling as well as a corresponding increase in cell death markers such as cleaved caspase 3 and cleaved PARP. Interestingly, there was a robust increase in VEGFR1 expression in the diabetic animals. However, treatment with recombinant VEGFB did not elicit downstream signaling as seen with control cardiomyocytes, suggesting a defect in VEGFR1 in the diabetic heart. These results (low VEGFB, increased VEGFR1 and blunted signaling) were also duplicated in animals made severely diabetic with 100 mg/kg STZ (D100) and monitored for 4 days. Interestingly, although insulin treatment of these D100 animals to produce euglycemia restored VEGFB protein expression, there was no change in VEGFR1 expression or its downstream signaling. These data for the first time suggested that the loss of VEGFB and its downstream signaling events is an early event after hyperglycemia, is sustained with disease progression, and could be added to the list of potential instigators that lead to cardiomyopathy in these T1D animals. In this regard, a reduction of VEGFB will be unable to withstand the forces like oxidative stress that are propelling the diabetic cardiomyocyte towards cell death. As cardiomyopathy is also a feature of Type 2 diabetes (66), the role of VEGFB in progression of heart dysfunction in animal models of T2D is also of interest and should be examined. In addition to cardiomyopathy, diabetic retinopathy is also a major consequence of diabetes and human retinal endothelial cells cultured in high glucose displayed decreased VEGFB gene expression (69). In rats made diabetic with 50 mg/kg STZ and injected with multiple intravitreal VEGFB injections 10 weeks later, TUNEL staining of the rat ganglion cell layer revealed a decrease in the number of apoptotic cells in the STZ animals treated with VEGFB (70).

### Therapeutic Options

During diabetes, the heart can no longer utilize glucose as an energy source and must adapt to use FA to generate ATP (5). While this switch ensures the heart is able to manage its constant energy demands, the increased reliance upon FA leads to a number of consequences. Generating ATP through FA oxidation requires more oxygen than using glucose and in diabetes there is blunted VEGFA mediated angiogenesis leading to a reduced supply of oxygen (6, 71). This lack of oxygen bottlenecks FAO resulting in the diabetic heart having to store the excess FA as triglycerides (72). Furthermore, the accumulation of triglycerides leads to the formation of ceramides and diacylglycerols which can lead to cardiac cell death (3). It is within this paradigm that the efficacy of VEGFB, as a therapeutic opportunity for the diabetic heart, is compelling. VEGFB has the capability of enhancing VEGFA induced angiogenesis (50) which may aid in providing the heart the necessary oxygen to metabolize the increased supply of FA. In addition, through its actions promoting cell survival, VEGFB could limit cardiac cell death and help delay heart failure. In conclusion, we suggest that using VEGFB as a cardio protective therapy in diabetes is an intriguing concept and should be explored.

### Limitations

Although the role of VEGFB in angiogenesis and cell death is an emerging topic, its connection to diabetes is in its infancy. Some studies have suggested that knockout of VEGFB promotes insulin sensitivity and decreased fatty acid accumulation (73, 74). However, these studies were all conducted in mice, and were not always repeatable (75). The beneficial effects of VEGFB have largely been obtained in transgenic rats and models of cell death in dogs and mice. Our own studies show that in an STZ model of Type 1 diabetes in rats, there is a robust decrease in VEGFB. Hence, when investigating the actions of VEGFB, it is critical that consideration should be given to the animal model being used. It should also be noted that one third of patients with diabetes show signs of diabetic retinopathy (76) and higher levels of VEGFB have been reported in the vitreous of patients with diabetic ocular disease (77). Hence, the therapeutic value of VEGFB in treating diabetic cardiomyopathy needs to be considered in relation to the possible accompaniment of retinopathy in these patients.

## Author Contributions

All authors listed have made substantial, direct, and intellectual contribution to the work and approved it for publication.

## Conflict of Interest Statement

The authors declare that the research was conducted in the absence of any commercial or financial relationships that could be construed as a potential conflict of interest.
